# Doctors, suicide and mental illness

**DOI:** 10.1192/bjb.2018.11

**Published:** 2018-08

**Authors:** Clare Gerada

**Affiliations:** National Health Service Physician Health Programme, London, UK

## Abstract

This article focuses on doctors and suicide. It provides real examples to illustrate why doctors die by their own hand. These reasons are replicated in the general population, but also include a host of additional risk factors related to being a doctor. In each case, information about the doctor is in the public domain or, as in one case, consent from the next of kin has been obtained for a detail not in the public domain. The author is a doctors' doctor, heading up a confidential health service for doctors with mental illness or addiction, the National Health Service Practitioner Health Programme. Mortality data from the programme (January 2008–January 2017) will also be included. For the sake of confidentiality, data is collated and details regarding age and gender have been approximated.

**Declaration of interest:**

None.

In most healthcare systems (whether privately or publicly funded), and across all ages, genders, specialties and seniority, doctors have higher rates of depression and anxiety compared with the general population and other professional groups.^1^ This is counterintuitive given that doctors have a host of apparently protective attributes, including career and financial security, high status and a generally rewarding job. However, doctors are as exposed as anyone else to risks associated with genetic predisposition, early traumatic life events, later bereavements, illnesses or relationship breakdowns. Doctors also have additional risk factors. They are chosen for personality traits that predict good doctoring – perfectionism, obsessiveness and even elements of martyrdom – traits that can act against them. From an early age they are driven, competitive, compulsive, individualistic and ambitious – features that can go into overdrive when stressed. As doctors work harder, they blame themselves for not being able to deliver the care required by their patients, and feel guilty for events beyond their control. Consequently, doctors can suffer from a triad of guilt, low self-esteem and a persistent sense of failure.^2^ To survive a lifetime in medicine, doctors also have to develop psychological defences that include depersonalisation and dissociation. This can make it harder to create attachments to others or to recognise when the emotional burden of their work becomes too much, and thus contributes to the spiralling of discontent and increased risk of suicide.

## Doctors and suicide

In the UK, around one in five adults has considered suicide and one in 15 have attempted it. Very few attempts result in death, estimated to be about 1 in 10 000 per annum.^3^ The suicide rate for doctors have been variably estimated at between two and five times the rate of the general population.^4^^,^^5^ In a systematic review, Lindeman *et al*. estimated physicians' relative suicide risk at 1.1–3.4 for men and 2.5–5.7 for women compared with those for the general population, and at 1.5–3.8 for men and 3.7–4.5 for women compared with those for other professionals.^6^ Anaesthetists, general practitioners and psychiatrists appear to be associated with higher risk. In an Australian survey, approximately a quarter of doctors reported having had thoughts of suicide prior to the past 12 months (24.8%), and 10.4% reported having had thoughts of suicide in the previous 12 months. Thoughts of suicide are significantly higher in doctors compared with the general population and other professionals (24.8 *v.* 13.3 *v.* 12.8%).^7^ In a 2008 study, members of the American College of Surgeons were sent an anonymous survey with questions on suicidal ideation and use of mental health services, and questionnaires for depression, burnout and quality of life.^8^ Of 7905 participating surgeons (a response rate of 31.7%), 501 (6.3%) reported suicidal ideation during the previous 12 months (more common in older surgeons). These levels of suicidal thoughts were between 1.5 and 3.0 times more common among surgeons than the general population. Importantly, only 26.0% of the surgeons with suicidal thoughts had sought help, whereas 60.1% (301) were reluctant to seek help because of concern that it could affect their medical license. This study shows the high rate of mental health distress accompanied by low use of treatment services.

## Suicide and mental illness

Major risk factors for completed suicide across all populations are depression and substance misuse, both of which are also common in doctors who take their own life. For example, Dr Louise Tebboth, a gifted south London general practitioner, was only 40 years old when she killed herself. Her husband Gary Marson, in his book *Carry on Breathing*,^9^ describes in poignant detail the first year of his bereavement, starting on Friday 23 January 2015 when he found her hanging from the bannisters in their home. She fought a long battle against bipolar disorder. She had survived a near fatal overdose in her late 20s but decades later, despite the intervention of psychiatric services and daily monitoring by her husband, and an intensive regime of activity to keep her occupied and safe, she killed herself.

## Deaths of doctors attending the National Health Service Practitioner Health Programme

As well as being a local general practitioner colleague, Louise was a patient of the National Health Service Practitioner Health Programme (PHP) when she died. Of the approximately 3500 doctors who have presented between 2008 and 2017, 80% have done so with mental health problems (mainly depression, anxiety and symptoms indistinguishable from post-traumatic stress disorder). Another 15% have predominantly suffered from alcohol or drug misuse (mostly alcohol dependence) or a mix thereof; the service also has a number of doctors with personality disorder, bipolar disorder, physical health problems affecting their mental health and a small number with undiagnosed schizophrenia or psychosis. During this period, 21 patients of the service have died; approximately 6 times as many men died as women. The average age at death was 44 years, ranging from under 30 to over 65 years old, and from early on in training to senior consultants near retirement. General practitioners accounted for 7 out of 21 (33%) of the deaths; the remainder were drawn from different medical, surgical, anaesthetic and emergency specialties in almost equal numbers. Six doctors died from suicide. A further ten died from accidents where the cause of death was not given as suicide, but which could be considered to be part of a suicidal act or from fatal overdoses of drugs/alcohol (not classified as suicide). Five were from either natural causes or the doctor died overseas and we have been unable to find the cause of death.

## Doctors, suicide and barriers to care

It is not just mental illness that predisposes doctors to killing themselves. Suicide is also linked to how doctors are treated, how they treat themselves, unique issues related to their job and a system where doctors with mental illness are handled through an adversarial rather than treatment system. This equates to personal, professional and institutional stigma, which doctors face when trying to access care and also once in treatment. Stigma is one of the most important barriers for doctors trying to be treated, illustrated by the next doctor. Daksha Emson was a young psychiatrist. On 9 October 2000, when her first child, Freya, was 3 months old, she stabbed her baby, poured accelerant on them both and set it alight. Both died. Daksha had written in her diary, just before her death, about her feelings of hopelessness. What follows is an abridged extract from that diary entry:

‘*Feel useless as a mother as a wife as a woman.*

See no hope for the future.

sleep unrefreshing food forced down because my baby needs nourishment. Focusing on my precious baby Freya – she means everything to me, I desperately want to be a good mother to her but I'm starting to feel I'm failing her in a big way, that everyone can see I'm a useless mother that I'm no good.

… hits me in early hours of morning – thoughts churn round and round.

*Finding it difficult to hang on to reality - am I bad and wicked? I don*'*t deserve good things, is there really hope for the future?*’

Extract from the last diary entry of Dr Daksha Emson, published in *Report of an Independent Inquiry into the Care and Treatment of Daksha Emson and her Daughter Freya*.^10^

The subsequent inquiry identified stigma as a major factor in her and her daughter's deaths. Daksha, like many doctors, felt that she had failed by becoming mentally unwell. The study by Henderson *et al*.^11^ of doctors out of work with mental illness found that most felt guilty, shamed and fearful. Doctors feel a dreadful sense of personal failure and inadequacy if they struggle to keep working and despair can be sudden and overwhelming. The researchers describe an overwhelming stigmatisation that mentally ill doctors were exposed to by friends, family and peers, which left them isolated and sad. Some sick doctors deliberately concealed their problems, and this resonates with doctors attending PHP who will pretend to go to work each day rather than admit to their families that they are unwell. In the Henderson *et al*. study, doctors described a lack of support from colleagues and feared a negative response when returning to work. Self-stigmatisation was central to the participants' accounts and several doctors appeared to have internalised the negative views of others. Stigma was also a key feature of a survey conducted by Cohen *et al*. of almost 2000 doctors.^12^ Cohen *et al*.'s view is that fewer professions stigmatise mental health disorders more prominently than medicine, a conclusion supported by the finding that 41% of doctors with mental illness said that they would not disclose it. Garelick *et al*.,^13^ reporting on the service for doctors with mental health problems, also cites stigma as a major barrier to receiving appropriate care.

Daksha was concerned about the stigma of her mental illness and its effect on her career progression; she mentioned this fear to her best friend several times. Even if, as with Daksha, doctors present for help, it is difficult for caregivers to see beyond the professional to the patient and treat the sick doctor as the frightened, vulnerable individual they are. Doctors tend to treat sick doctors differently from other patients. They engage in medical talk, discuss academic papers or the latest research and go way beyond what the sick doctor really wants or needs. The PHP discourages shared decision-making until the sick health professional begins to improve. Daksha's untimely death led to funding for PHP, with which thousands of doctors with mental illness have been helped.

## Doctors, suicide and complaints

Dr Wendy Potts was a 46-year-old mother of two and a general practitioner in Derbyshire. She also suffered from bipolar disorder. Dr Potts wrote about her experience with depression on an online blog. In it, she wrote openly on how fluctuations in her mood affected her and her life in general. It is reported that a patient complained after reading her blog, questioning whether she should be able to practise as a general practitioner. She was suspended by her practice and National Health Service (NHS) England, and referred to the General Medical Council (GMC). In November 2015, Dr Potts hung herself. Dr Potts' case incorporates both stigma and mental illness, but also the additional burden that weighs heavily on doctors and adds to their risk – complaints and disciplinary processes. Bourne *et al*.^14^ conducted a study of doctors comparing the mental health (using standardized tools) of those who had and had not received a complaint. A total of 10 930 out of 95 636 (11.4%) responded, and 7926 (8.3%) completed the full survey and were included in the complete analysis. Of those who completed the survey, 16.9% of doctors with current or recent complaints reported moderate/severe depression (relative risk 1.77; 95% CI 1.48–2.13) compared with doctors with no complaints (9.5%). A total of 15% reported moderate/severe anxiety (relative risk 2.08; 95% CI 1.61–2.68) compared with doctors with no complaints (7.3%). The authors found that distress increased with complaint severity, with highest levels after a GMC referral (26.3% depression, 22.3% anxiety). Doctors with current or recent complaints were 2.08 (95% CI 1.61–2.68) times more likely to report thoughts of self-harm or suicidal ideation. This analysis illustrates how damaging complaints and regulatory processes are to doctors’ health and job performance. Similar findings of the negative effect of disciplinary processes on the mental health of doctors were found in a study from the Netherlands.^15^ Analyses of the deaths of doctors at PHP show a significant correlation between mortality and involvement of the regulator. Among PHP patients who have not died, the GMC is involved in around 10% of cases, compared with 11 out of 21 (52%) of patients who have died and 9 out of 16 (56%) patients who died from accidents, suicide or overdoses (Table 1).

**Table 1:** Analysis of patients seen at the National Health Service Practitioner Health Programme who have died, and their involvement with General Medical Council (GMC) procedures

Cause of death	Number of patients	GMC involvement
All deaths	21	11
Overdose drugs/alcohol or accidents	10	9
Suicide	6	

In response to concerns about high numbers of deaths among doctors, the GMC commissioned an independent study examining 28 deaths of doctors due to suicide (or suspected suicide) where the doctor was also involved in fitness-to-practise processes between 2005 and 2013.^16^ The case reviews of doctors during this period showed that many of the doctors who died by suicide suffered from a recognised mental health disorder or had drug and/or alcohol addictions. Other factors that often followed from those conditions may have also contributed to their deaths. These include marriage breakdown, financial hardship and in some cases, police involvement, as well as the stress of being investigated by the GMC. Of course, correlation between death and regulatory involvement does not equate to causation, as the GMC review found. The interaction between complaints and mental illness is complex, with many possible issues to take into account (Table 2).

**Table 2:** Relationship between regulatory processes and mental illness in doctors

Relationship between suicide in doctors and complaints/regulatory or disciplinary processes
• A complaint may lead to a doctor becoming depressed or worsen a pre-existing mental illness. • Mental illness can lead to cognitive impairment, which can lead to a boundary transgression or inappropriate behaviour such as bullying or acting inappropriately with a patient or work colleague. • Mental illness might lead to out-of-character criminal behaviour (such as shoplifting), which itself can lead to worsening of the mental illness. • Mental illness might involve criminal activity; for example, drug use. • Drug use can lead doctors transgressing good medical practice, such as stealing drugs, self-prescribing or prescribing in a patient's name for the doctor's own use. • Mental illness in itself might be considered counter to fitness-to-practise; for example, bipolar disorder, schizophrenia, personality disorder or schizoaffective disorder. • The very act of trying to kill oneself might lead to criminal or professional sanctions where the means of the suicide attempt involves obtaining drugs illegally or via self-prescription.

At Dr Potts' inquest, the coroner commented that the system had lost sight of the fact that there was a human being behind the complaint and investigation.^17^ It is indeed common to underestimate the effect that complaints can have on doctors, and to lose site of the severe pain this causes to the doctor and how a complaint can threaten a doctor's sense of self. A complaint challenges a doctor's values. It is a catastrophic personal event, described by one doctor at PHP as akin to a diagnosis of cancer. The overwhelming feeling (once the anger and shock as subsided) is that of shame: shame of disclosure, of appearing in front of the regulator, of having to face the gauntlet of the press and the shame brought on their families, friends and colleagues. All too often, their shame becomes exaggerated and they begin to feel responsible for the entire profession's values and future.

## Prevention

Preventing a very rare event (completed suicide) and identifying those who will go on to complete a suicide act from those who express suicidal thoughts is extremely difficult, if not impossible. A systematic review of risk assessment for suicide by Large *et al*.^18^ concluded that the overwhelming majority of people who might be viewed as high risk for suicide will not kill themselves, and about half of all suicides will occur among people viewed as low risk. Carter *et al*.^19^ found similar results in their systematic review of instruments aimed at predicting high risk of suicide and concluded that no high-risk instrument was clinically useful. This is what we have found among our doctors at PHP. PHP risk-assesses all patients at first assessment and reviews thereafter as required. Patients are risk-assessed depending on the perceived risk to self, service/institution or their own patients. This assessment forms part of the weekly multidisciplinary team meeting. Only 3 of the 21 doctors who died were assessed as being high risk (red) (recent/current suicidal ideation, past attempt to take one's own life, drug misuse, alcohol dependence or depression are all risk factors), and most (16 out of 21) were considered by the service as low (green) risk. Two doctors who killed themselves were rated red (the highest risk), and the other doctor died from an overdose of drugs.

## Conclusion

It is important to remember that the vast majority of doctors do not kill themselves. Most doctors thrive in their working environment. However, each death is a tragedy which sends repercussions through the system and poses the risk of creating contagion. Going forward, we have to halt the decline in morale among doctors. This will mean addressing many systemic issues that are creating unhappiness: tackling the culture of naming, blaming and shaming and the constant denigration of NHS staff by the press; allowing doctors to maintain a sensible work–life balance and not ignoring the basic needs of staff who give their all to their patients. We must restore doctors' collective self-esteem by treating them as intelligent adults and not naughty schoolchildren, and by creating a culture in which their skills can flourish. We need to ensure doctors have access to early intervention and confidential support services.^20^ Finally, we have to ensure that all NHS staff receive the same compassion that they, rightly, are expected to give to their patients.

## About the author

**Dr Clare Gerada** is the Medical Director of the National Health Service Practitioner Health Programme, London.
